# Inhibition of HDAC6 alleviating lipopolysaccharide-induced p38MAPK phosphorylation and neuroinflammation in mice

**DOI:** 10.1080/13880209.2018.1563620

**Published:** 2019-05-24

**Authors:** Yuanjian Song, Li Qin, Rongli Yang, Fan Yang, Nwobodo Alexander Kenechukwu, Xiaofang Zhao, Xiaoyan Zhou, Xiangru Wen, Lei Li

**Affiliations:** a Department of Genetics, Xuzhou Medical University, Xuzhou, Jiangsu, PR China;; b Jiangsu Key Laboratory of Brain Disease Bioinformation, Xuzhou Medical University, Xuzhou, Jiangsu, PR China;; c Department of Geriatrics, Affiliated Hospital of Xuzhou Medical University, Xuzhou, Jiangsu, PR China;; d Laboratory of Morphology, Xuzhou Medical University, Xuzhou, Jiangsu, PR China

**Keywords:** Histone deacetylase 6, Tubastatin A, inflammation, phosphorylated p38MAPK

## Abstract

**Context:** Researchers in a variety of fields have extensively focused on histone deacetylase 6 (HDAC6) due to its aggravation of inflammatory reaction. However, relevant studies examining whether HDAC6 could exacerbate lipopolysaccharide (LPS)-induced inflammation are still lacking.

**Objective:** We assessed the role of HDAC6 in LPS-induced brain inflammation and used the HDAC6-selective inhibitor Tubastatin A (TBSA) to investigate the potential mechanisms further.

**Materials and methods:** Brain inflammation was induced in Kunming (KM) mice via intraperitoneal (I.P.), injection of Lipopolysaccharide (LPS) (1 mg/kg), the TBSA (0.5 mg/kg) was delivered via intraperitoneal. The phosphorylated p38 (p-p38) Mitogen-activated protein kinases (MAPK) and expression of typical inflammatory mediators, including tumor necrosis factor-α (TNF-α) and interleukin-6 (IL-6) in both the hippocampus and cortex, were examined by immunoblotting. Nissl staining was used to detect the neuronal damage in the hippocampus and the cortex.

**Results:** About 1 mg/kg LPS via daily intraperitoneal (I.P.) injections for 12 days significantly increased p38 MAPK phosphorylation, TNF-α and IL-6 expression, and neuronal loss. However, 0.5 mg/kg TBSA (three days before LPS treatment) by I.P. injections for 15 days could reverse the above results.

**Conclusions:** This present study provided evidence that TBSA significantly suppressed LPS-induced neuroinflammation and the expression of p-p38. Results derived from our study might help reveal the effective targeting strategies of LPS-induced brain inflammation through inhibiting HDAC6.

## Introduction

Inflammation may be a key factor regarding the pathogenesis of brain injury and neurodegenerative disorders, such as Alzheimer’s disease (AD) and Parkinson’s disease (PD) (Ding et al. 2017; Huang et al. 2017; Schwenkgrub et al. 2017). Hippocampus and cortex are the two susceptible regions in inflammatory damage in the brain (Del et al. 2011; Jiang et al. 2017). Lipopolysaccharide (LPS) is a bacterial endotoxin that interacts with p38 mitogen-activated protein kinase (p38 MAPK) receptors on macrophages and triggers inflammatory responses and p38MAPK has well-established functions in the LPS-mediated inflammatory response (Zhong et al. 2016). Furthermore, the role of p38MAPK in neuronal damage has been confirmed following the administration of relatively high concentrations of LPS in the brain (Wu et al. 2016). HDAC6 overexpression regulates the inflammatory response by activating MAPK species, including p38MAPK, ERK and JNK (Ahmed et al. 2013; Youn et al. 2016). However, the relationship between HDAC6 and p38MAPK in LPS-injected mice remains unknown. We hypothesized that HDAC6 accelerates LPS-induced brain injury by increasing the production of pro-inflammatory factors and enhancing p38 MAPK phosphorylation.

Studies have shown that LPS induces the production of pro-inflammatory cytokines, including interleukin-6 (IL-6) and tumor necrosis factor-alpha (TNF-α), which affects the brain function (Zhang et al. 2017). According to recent reports, peripherally injected LPS directly induces microglial activation and brain injury in rats. Therefore, intraperitoneal (I.P.) administered LPS can be used to prepare animal models of brain injury with inflammation. No reliable drugs are available for these neurodegenerative diseases (Fu et al. 2015). Though it has been reported that HDAC6 may emerge as a promising therapeutic target, genetic and pharmacological inhibition of HDAC6 have been shown to suppress neurodegenerative disorders, enhance immunomodulatory activity and alleviate disorder behaviors in animal models (Jianhua et al. 2017; Martin et al. 2017). Also, suppression of HDAC6 activity significantly restrains the LPS-induced production of pro-inflammatory cytokines (Yoo et al. 2016). The anti-inflammatory action of the HDAC6-selective inhibitor Tubastatin A (TBSA) has recently been shown to decrease inflammation of rat thermal injury (Shen et al. 2015). Thus, TBSA is an ideal drug for some inflammatory diseases in the brain. However, the molecular mechanisms of HDAC6 in brain diseases require further studies. In the present study, we examined the effect of HDAC6 on the phosphorylation of p38MAPK and the expression of TNF-α and IL-6 to investigate whether p38MAPK was involved in the HDAC6-relative neuroinflammation induced by LPS.

## Materials and methods

### Animals

One-month-old male KunMing (KM) mice weighing 29 ± 3 g, were purchased from the Shanghai Experimental Animal Center (Chinese Academy of Sciences), had *ad libitum* access to water and rodent chow, and were maintained at 21 °C under a reverse-phase 12 h light/dark cycle. All surgeries were performed under chloral hydrate anesthesia and all efforts were made to minimize suffering in accordance with the guidelines for the care and use of laboratory animals and were approved by the local committee for the ethics of animal experimentation.

### Reagents and antibodies

LPS (from *Salmonella typhimurium*) and the HDAC6-specific inhibitor TBSA were purchased from Sigma (St. Louis, MO, USA). Rabbit monoclonal anti-phosphorylated p38MAPK and anti-IL-6 antibodies were also purchased from Santa Cruz Biotechnology. Rabbit monoclonal anti-TNF-α antibody was bought from Abcam (UK). The secondary antibody used in our experiment was goat anti-rabbit IgG from Sigma.

### Experimental procedure and drug administration

**Figure 1.**
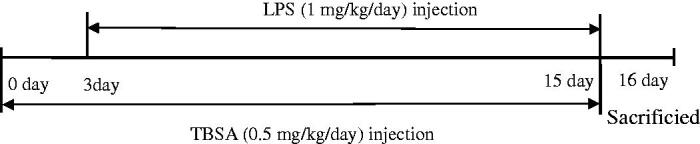
Schematic overview of the experimental procedure used in the present study.

Mice were divided into three groups, NS group, LPS group, and TBSA group. LPS (mg/mL) was dissolved in sterile saline as a stock solution. The TBSA was dissolved in 1% dimethyl sulfoxide (DMSO). LPS and TBSA groups were administered 1 mg/kg LPS via daily intraperitoneal (I.P.) injections for 12 days, TBSA group received 0.5 mg/kg TBSA (three days before LPS treatment) by I.P. injections for 15 days; the NS group only received an equal volume of 0.9% saline (the experimental process is shown in Figure 1).

### Sample preparation

The mice were anesthetized with chloral hydrate (350 mg/kg, I.P.) 24 h after LPS and TBSA last administration and immediately decapitated. For molecular analyses, the whole brains were removed for dissection, and the hippocampi and the frontal cortex were separated and quickly frozen in liquid nitrogen. Tissue samples were homogenized in ice-cold homogenization buffer containing 10 mm HEPES (pH 7.9), 1 mm DTT, 1 mm Na_3_VO_4_, 1 mm 4-nitrophenyl phosphate (PNPP) and enzyme inhibitors (0.5 mg/mL PMSF and 10 g/mL each of pepstatin A, leupeptin, and aprotinin). Afterward, the samples were centrifuged at 14,000 g for 15 min at 4 °C. The supernatants were carefully collected and stored at −80 °C until use. The protein concentrations were determined using a BCA assay kit.

### Immunoblotting

Immunoblotting analyses were conducted by separating the proteins on 10–12.5% SDS-PAGE gels. The proteins (80 mg) were then electrotransferred onto a nitrocellulose membrane (NC). After blocking for 2 h in Tris-buffer with 0.1% Tween-20 (TBST) and 3% bovine serum albumin (BSA), the membranes were incubated with primary antibodies (1:200) in TBST containing 3% BSA overnight at 4 °C. The membranes were washed and incubated with secondary antibodies (1:200) in TBST for 2 h. The densities of the bands on the membrane were scanned and analyzed using image analysis software (Quantity One, Bio-Rad Company, Hercules, California, USA).

### Histology

The remaining mice were anesthetized and perfused with 4% paraformaldehyde in 0.1 M sodium phosphate buffer (pH 7.4). Brains were separated quickly and post-fixed overnight in a fixation solution at 4 °C, followed by processing and embedding in paraffin. Coronal sections (5 μm thick) were cut on a microtome. Sections were deparaffinized with xylene and rehydrated with a gradient of ethanol solutions and distilled water. The sections were stained with 0.1% cresyl violet and the surviving neurons in the hippocampus and frontal cortex were observed under a light microscope (magnification × 200). The number of surviving hippocampal neurons and frontal cortical neurons per 1 mm^2^ area were counted to determine the neuronal density. Six mice from each group were analyzed.

### Statistical analysis

At least six independent animals were sampled for the immunoblotting and cresyl violet stain. A semi-quantitative analysis of the bands was performed using the Image J analysis software. All values were expressed as means ± SEM. The results were statistically analyzed using a one-way analysis of variance (ANOVA), followed by a least significant difference (LSD) test or a Newman–Keuls test. *p* Values <0.05 were considered significant.

## Results

### Inhibition of HDAC6 suppresses p38MAPK phosphorylation

**Figure 2.**
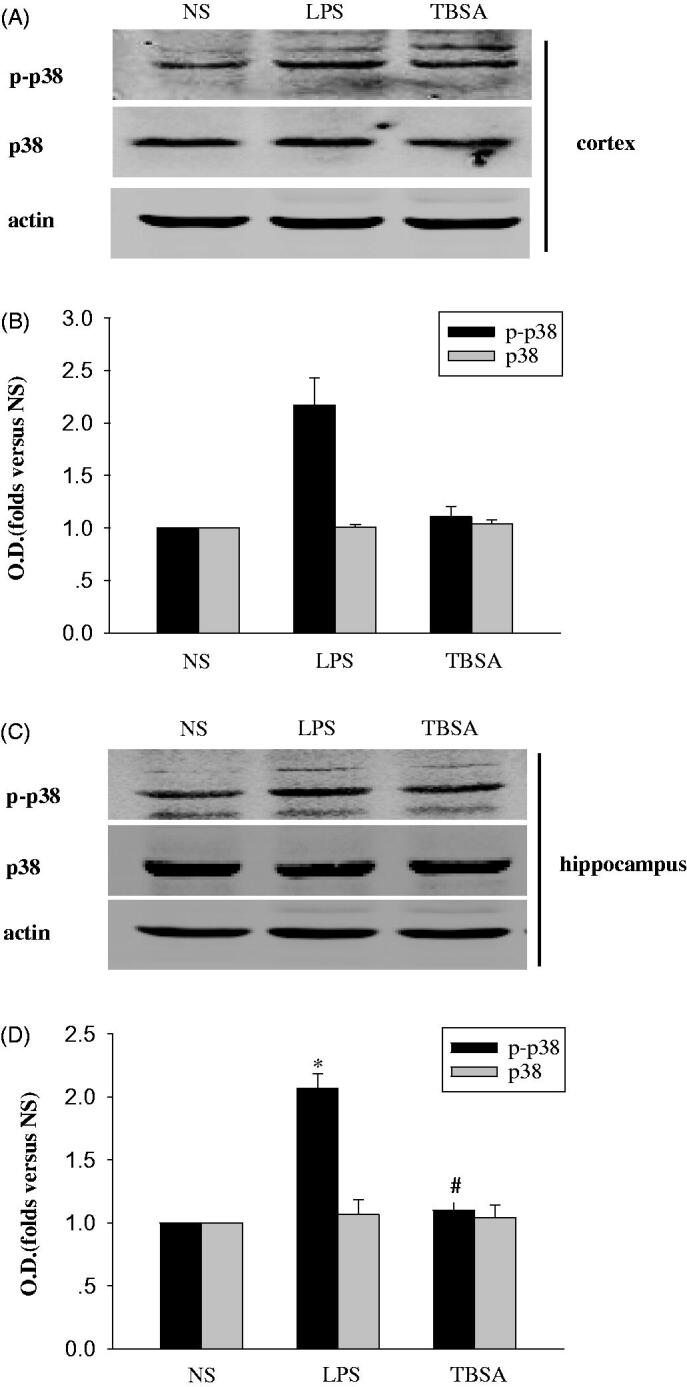
HDAC6 inhibition suppresses p38MAPK expression in the mouse frontal cortex and hippocampus after LPS treatment. (A and C) Immunoblots of the phosphorylation of p38MAPK in the cortex and hippocampus. (B and D) The intensity of the bands was determined by analyzing the optical density (O.D.). Data are presented as means ± SEM and are expressed as fold changes compared with the saline group. *p < 0.05 compared with respective NS group; #*p* < 0.05 compared with respective LPS group (*n* = 6).

HDAC6 overexpression activates p38MAPK to regulate the inflammatory response in macrophages (Youn et al. 2016). We selected LPS injections to induce chronic systemic inflammation and determined whether phosphorylation of p38MAPK in the hippocampus and cortex were involved in brain injury and to verify whether the inhibition of HDAC6 would suppress p38 MAPK phosphorylation in the presence of LPS-induced neuroinflammation. Immunoblotting was used to detect the effect of the HDAC6 inhibitor (TBSA) on the phosphorylation of p38MAPK. Compared to the NS group, p38 MAPK phosphorylation in LPS group was significantly increased in the cortex and hippocampus. However, TBSA treatment could reverse these increases in the TBSA group (Figure 2). Therefore, TBSA might inhibit the phosphorylation of p38MAPK in response to LPS.

### Inhibition of HDAC6 restrains TNF-α and IL-6

**Figure 3.**
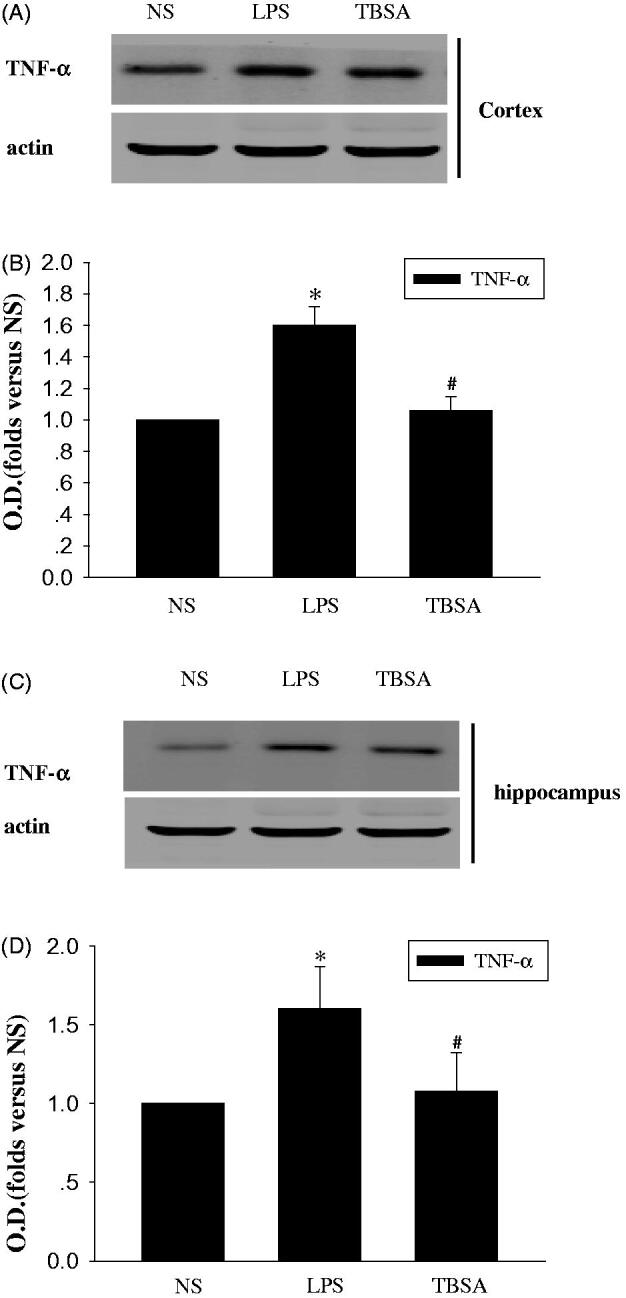
HDAC6 inhibition restrains the expression of TNF-α in the frontal cortex and hippocampus after LPS treatment. (A and C) Immunoblots of the levels of the TNF-α protein in cortex and hippocampus. (B and D) The intensity of the bands was determined by analyzing the optical density (O.D.). Data are presented as means ± SEM and are expressed as fold changes compared with the saline group. *p < 0.05 compared with respective NS group; #*P* < 0.05 compared with respective LPS group (*n* = 6).

**Figure 4.**
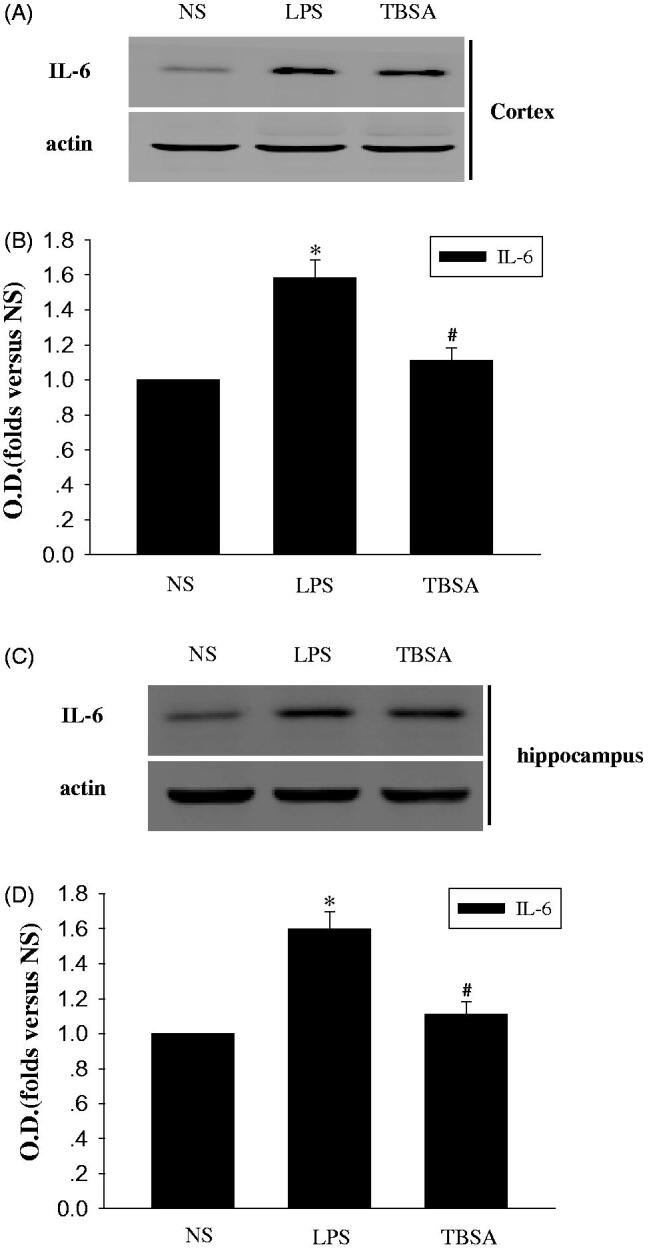
HDAC6 inhibition restrains the expression of IL-6 in the frontal cortex and hippocampus after LPS treatment. (A and C) Immunoblots of the levels of the IL-6 protein in cortex and hippocampus. (B and D) The intensity of the bands was determined by analyzing the optical density (O.D.). Data are presented as means ± SEM and are expressed as fold changes compared with the NS group. *p < 0.05 compared with the NS group; #*p* < 0.05 compared with the LPS group (*n* = 6).

The actions of the cytokines, such as TNF-α and IL-6 involved in chronic inflammation contributes to the response of the brain to LPS (Yang et al. 2017). Next, we examined the pro-inflammatory response in the hippocampus and cortex. Immunoblotting was used to detect the effect of TBSA on the expression of TNF-α and IL-6. As shown in Figures 3 and 4, the expression of TNF-α and IL-6 were significantly higher in the LPS group than in the NC group and TBSA decreased the expression of TNF-α and IL-6 in the cortex and hippocampus of LPS-treated mice.

### Inhibition of HDAC6 diminishes neuronal damage

**Figure 5.**
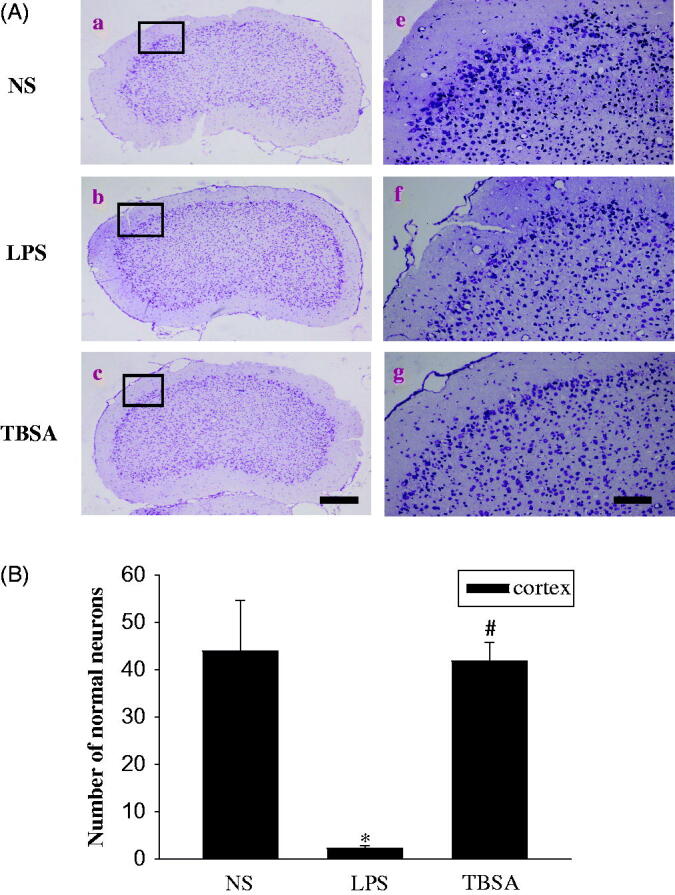
HDAC6 inhibition diminishes neuronal damage in the frontal cortex. Typical images of cresyl violet-stained sections from the frontal cortex of the NS group (a, d), mice injected with LPS (b, e) and mice treated with both LPS and TBSA (c, f). Data were obtained from six independent animals in each experimental group and the results of a typical experiment are presented here. Boxed areas in the left column are shown at higher magnification in the right column. Scale bar in d = 200 μm; Scale bar in h = 20 μm. (B) The cell density in the frontal cortex was calculated. Data were obtained from six independent animals in each experiment group. *p < 0.05 compared with the respective saline group; #*p* < 0.05 compared with the respective LPS group.

**Figure 6.**
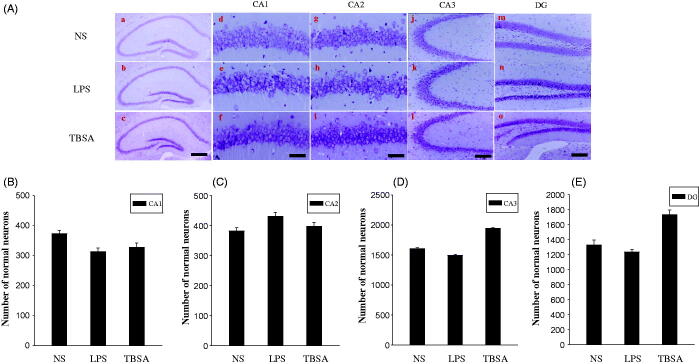
Effect of HDAC6 inhibition on neurons in the hippocampus. (A) Typical images of cresyl violet-stained sections from the hippocampus of the NS group (a, d, g, i and m), mice injected with LPS for 12 days (b, e, h, k and n) and mice treated with both LPS and TBSA (c, f, i, l and o). Data were obtained from six independent animals in each experimental group and the results of a typical experiment are presented here. Boxed areas in the left column are shown at higher magnification in the right column. Scale bar in d = 200 μm; Scale bar in h = 20 μm. (B, C, D and E) The cell density was calculated in the hippocampal CA1, CA2, CA3 and DG regions. Data were obtained from six independent animals in each experiment group. *p < 0.05 compared with the respective saline group; #*p* < 0.05 compared with the respective LPS group.

Cresyl violet staining was used to examine the surviving neurons in the frontal cortex and hippocampus. Normal cells present round and pale stains nuclei, whereas the normal morphological structure disappears in the damaged cells (Zhou et al. 2017). As shown in Figure 5, cells in the frontal cortex and hippocampus of the NS group showed round and pale stained nuclei, whereas the LPS group showed severe cell injury. TBSA group showed a significant decrease in neuronal loss in the frontal cortex compared to the LPS group. The neuroprotective effects of the TBSA treatment against LPS-induced neuronal damage and inflammation were statistically significant in the frontal cortex, but not in hippocampal subfields (Figure 6). These results indicated that inhibition of HDAC6 enhanced neuronal survival in the frontal cortex in LPS-treated mice.

## Discussion

In this study, we examined the vital role of TBSA in the biochemical changes observed in neurons from the frontal cortex and hippocampus of LPS-induced inflammation mice model. From the data, we could determine that TBSA treatment reduced the phosphorylated p38MAPK and the expression of TNF-α and IL-6 in the cortex of LPS-treated mice.

MAPKs are a family of evolutionally conserved molecules that play critical roles in cell signaling and gene expression and the MAPK family are made up of three significant members, including p38MAPK, extracellular signal-regulated kinase (ERK), and c-Jun N-terminal kinase (JNK) that are involved in three different signaling cascades (Che et al. 2017; Li et al. 2017). Also, p38MAPK is expressed at relatively high levels in the brain and plays a role in neuronal damage, regarding as a stress-induced kinase that plays a critical role in inflammatory responses (Wang et al. 2017). Inhibition of HDAC6 has been suggested to protect cells from LPS-induced injuries during the inflammatory response (Joshi et al. 2015; Yoo et al. 2016). We proposed that suppressing HDAC6 expression might be therapeutically useful during brain injury associated with chronic inflammation. Furthermore, HDAC6 overexpression regulates the inflammatory response by activating MAPK species, including p38MAPK, ERK and JNK in macrophages. Consistent with these findings, phosphorylation of p38MAPK was significantly elevated during LPS-induced inflammation. However, HDAC6 selective inhibitor TBSA treatment could decrease p38MAPK phosphorylation in the hippocampus and cortex. Hence, TBSA might suppress brain inflammation, which was involved in p38 MAPK.

In the brain, the inflammatory response to the LPS challenge is related to the release of pro-inflammatory cytokines, such as TNF-α and IL-6 (Yoo et al. 2016; Hedde et al. 2017). Therefore, strategies that suppress their production could protect against LPS-induced brain injury (Hedde et al. 2017). In the present study, LPS induced high expression of TNF-α and IL-6 in the cortex and hippocampus. Meanwhile, in comparison with the LPS groups, a significant reduction in TNF-α and IL-6 expression was observed in the cortex and hippocampus regions 15 days after TBSA treatment. Therefore, our findings support the hypothesis that HDAC6 is an essential factor that is primarily responsible for p38 MAPK phosphorylation in the LPS-induced neuronal injury. Our results also support the hypothesis that decreased HDAC6 expression protects against brain damage caused by inflammation. Together, HDAC6 exerted harmful effects on LPS-treated brains, which is related to the p38MAPK phosphorylation and the acceleration of TNF-α and IL-6 production. HDAC6 up-regulated the phosphorylation of p38MAPK by activating inflammatory mechanisms.

Based on the mounting evidence, the hippocampus and frontal cortex damage plays an important role in LPS-induced brain injury (Khan et al. 2017). In the present experiments, we employed cresyl violet staining to detect the survival of neurons in the hippocampus and frontal cortex to study the effect of TBSA treatment on neurons in LPS-treated mice. Neuronal density and survival were remarkably increased in the frontal cortex. Intriguingly, the healthy neurons in the hippocampal subfields were not significantly increased after TBSA treatment, the precise mechanisms underlying this phenomenon need to be deeply investigated in our further study. TBSA exerted a neuroprotective effect on neuronal injury in LPS-treated mice and was associated with the reduction of cellular damage in the frontal cortex.

In conclusion, HDAC6 selected inhibitor TBSA protected the neuronal cell against LPS-induced brain injury by suppressing p38MAPK signal pathway and inflammation in the frontal cortex and the hippocampus. Further studies are required to identify the detailed signaling mechanisms. The results of the present study demonstrated that TBSA might have a beneficial therapeutic value for the treatment of neuroinflammation. In addition, TBSA is suitable for long-term administration and may have potential therapeutic value for further clinical treatment of other neurological complications in patients with brain inflammation.
